# Complete mitochondrial genome of *Pelecanus crispus* and its phylogeny

**DOI:** 10.1080/23802359.2019.1666679

**Published:** 2019-09-19

**Authors:** Tian Huang, Yonghong Wang, Libo Zhou, Zhenggang Xu

**Affiliations:** a College of Information and Electronic Engineering, Hunan City University, Yiyang, China;; b Hunan Engineering Research Center for Internet of Animals, Changsha, China;; c Key Laboratory of Forestry Remote Sensing Based Big Data and Ecological Security for Hunan Province, Central South University of Forestry and Technology, Changsha, China

**Keywords:** *Pelecanus crispus*, Pelecaniformes, mitochondrial genome, phylogeny

## Abstract

The Dalmatian Pelican (*Pelecanus crispus*), in the order Pelecaniformes, its habitat is distributed in Europe, Asia, and Africa. It is a large waterfowl that is of international concern. In this study, we first sequenced and described the complete mitochondrial genome and phylogeny of *P. crispus*. The results showed that the whole genome of *P. crispus* was 16,131 bp in length, and contained 13 protein-coding genes, 22 transfer RNA genes, two ribosome RNA genes, and one non-coding control region. The overall base composition of the mitochondrial DNA was 30.48% for A, 23.15% for T, 31.68% for C, and 14.69% for G, with a GC content of 46.37%. A phylogenetic tree of *P. crispus* in Pelecaniformes confirmed that *P. crispus* was sister to *C. occidentalis*. This information will be useful in the current understanding of the phylogeny and evolution of Pelecaniformes.

The Dalmatian Pelican (*Pelecanus crispus*), which belongs to the Pelecaniformes. Its habitat is widely distributed in lakes, rivers, coastal waters, and tropical communities in Europe, Asia, and Africa (Shi et al. 2008). In recent years, due to the large reduction in population, it is listed as of Near Threatened (NT) on the IUCN Red List of Threated Species (IUCN 2017), and a secondary protected animal in China. Despite this, genetic information of *P. crispus* is quite limited, which lead to confused and contentious phylogenetic relationships among Pelecaniformes, Suliformes, and Ciconiiformes (Zhang et al. 2012). Therefore, it is necessary to sequence the complete mitochondrial genome of *P. crispus* to enhance our understanding of the phylogeny and evolution of Pelecaniformes.

The specimen was collected from Dongting Lake (29.407E, 112.969N) and stored at Hunan Engineering Research Center for Internet of Animals, China with accession number 20170707PC. The whole mitochondrial DNA was extracted from muscle specimen with DNeasy Plant Mini kit (Qiagen, Valencia, CA). The genomic DNA data were sequenced using Illumina Miseq platform (Illumina, San Diego, CA). The adapter and low-quality reads were filtered out using NGS QC toolkit (Patel and Mukesh 2012). The genome was annotated using the MITOS online service (Bernt et al. 2013). Annotated PCGS were compared with other vertebrate species sequences that have been published in DOGMA (Wyman et al. 2004). The circular map of the mitochondria was presented using OGDRAW (Lohse et al. 2007); it referred to Zhang’s method (Zhang et al. 2017). The complete mitochondrial genome of P. *crispus* has been submitted to the NCBI database with the accession number of MK855120. Phylogenetic tree of the relationships among Pelecaniformes and its related orders were presented using complete mitochondrial genome among 26 pelecaniformes species using neighbour-joining analyses in NCBI website with 1000 bootstrap replicates (Kumar et al. 2016). There is only one complete mitochondrial genome of *pelecanus* species, *P. occidentalis*, been reported (Huang et al. 2018).

The complete mitochondrial genome was 16,131 bp in length. It consists of 37 mitochondrial genes, including 13 protein-coding genes (PCGs), 22 transfer RNA (tRNA) genes, two ribosomal RNA (rRNA) genes, and one non-coding control region (D-loop). Among these genes, *ND6* and eight tRNAs (*trn^Gln^*, *trn^Ala^*, *trn^Asn^*, *trn^Cys^*, *trn^Tyr^*, *trn^Ser^*, *trn^Pro^*, and *trn^Glu^*) were located on the light strand, whereas other genes were located on the heavy strand. The *P. crispus* mitogenome consists 30.48% A, 23.15% T, 31.68% C, and 14.69% G, with a GC content of 46.37%. The reconstructed phylogenetic tree supported the placement of *P. crispus* in Pelecaniformes. All of the nodes were inferred with strong support using the NJ analysis. Our results confirmed that *P. crispus* was sister to *C. occidentalis* (Figure 1). In all, the mitochondrial genome reported here would be useful in the current understanding of the phylogeny and evolution of Pelecaniformes. We must pay more attention on *pelecanus* species for lacking genetic information.

**Figure 1.**
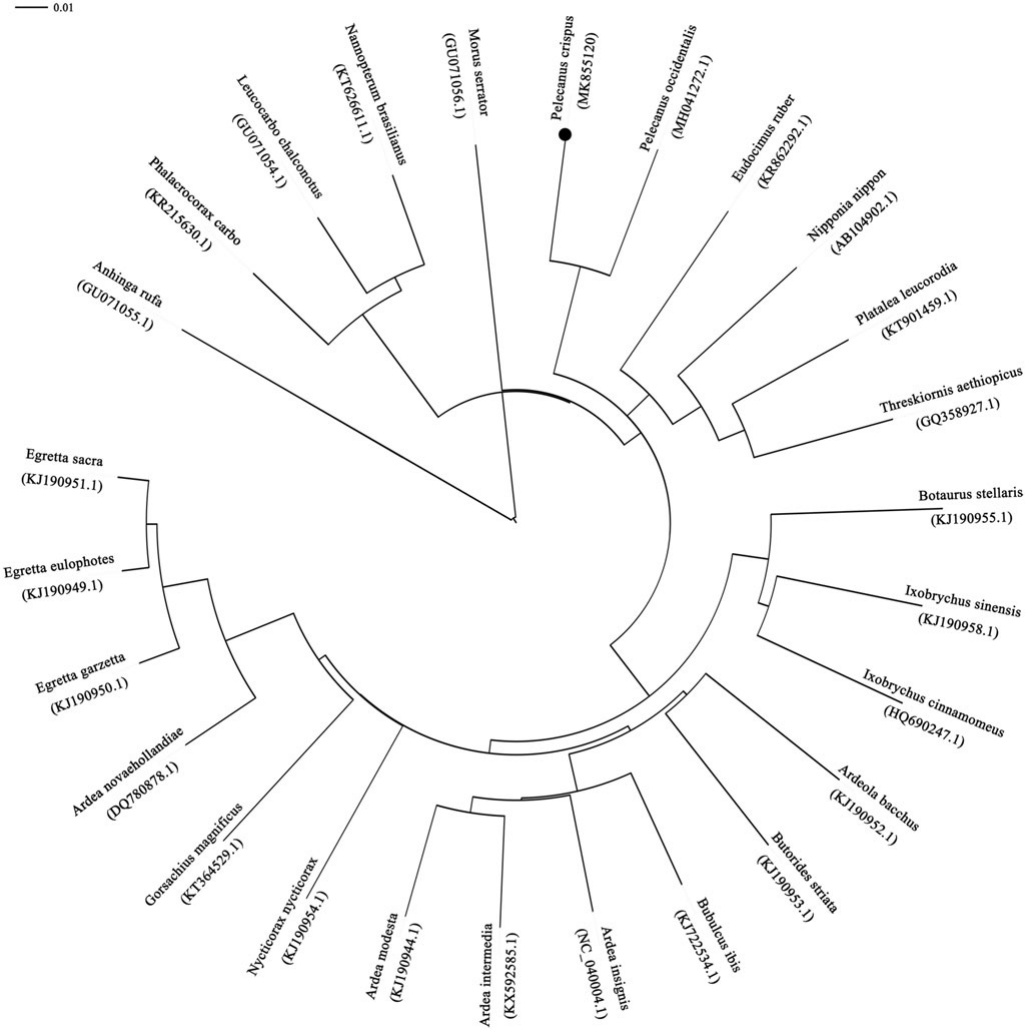
Phylogenetic tree of the relationships among Pelecaniformes and its related orders based on complete mitochondrial genome. Branch lengths and topologies came from the neighbour-joining (NJ) analyses.
